# Miliary tuberculosis occurred after immunosuppressive drug in PNH patient with completely cured tuberculosis; a case report

**DOI:** 10.1186/1476-0711-11-12

**Published:** 2012-05-03

**Authors:** Jihyun Lee, Soojung Gong, Byounghoon Lee, Soyoung Lee, Jungae Lee, Naeyu Kim

**Affiliations:** 1 Department of Internal Medicine, Eulji University College of Medicine, Eulji Medical Center, 14 Hangeulbiseok-gil, Nowon-gu, Seoul, 139-872, South Korea; 2 Department of Internal Medicine, Eulji University College of Medicine, Eulji Medical Center, Dunsan-dong, Seo-gu, Daejeon, 1306, South Korea

**Keywords:** Paroxysmal nocturnal hemoglobinuria, Miliary tuberculosis (TB), Tuberculosis (TB) prophylaxis

## Abstract

Paroxysmal nocturnal hemoglobinuria (PNH) is a clonal disorder that presents with hemolytic anemia, marrow failure and thrombophilia. During acute attacks, corticosteroid can alleviate the hemolytic paroxysm, but the prolonged administration induces serious toxicity including immunosuppression. So American thoracic society (ATS) for tuberculosis (TB) recommends prophylactic anti-TB medication in patients with a long-term steroid therapy. However, in the patient who was treated for active TB in the past, there are no guidelines of the test for determining patients who have latent TB infection (LTBI) and no recommendations of TB prophylaxis if there is no evidence of reactivation at present. A 40-year-old male patient presented with fever and aggravated weakness for a week. He was diagnosed with PNH a month ago and took corticosteroid for 3 weeks. In the past, he was diagnosed with pulmonary TB and completely cured after treatment. According to guideline, he was not indicated with TB prophylaxis. However, he caught miliary TB, progressed to acute respiratory distress syndrome. We experience this embarrassing case, and emphasize the need to investigate multicentral TB prevalence and to make the guidelines of anti-TB medication in subgroups of hematologic diseases including PNH.

## Introduction

Paroxysmal nocturnal hemoglobinuria (PNH) is a rare acquired clonal hematopoietic stem cell disorder that presents with hemolytic anemia, venous thrombosis, deficient hematopoiesis and chronic renal disease [1]. It is a serious chronic disease with high morbidity and mortality. Hematopoietic stem cell transplantation is only a curative treatment. The treatment is personalized and directed towards the specific complications. Sometimes, PNH patients have acute attacks. Corticosteroid and androgen can be lessen a hemolytic paroxysm and improve hemoglobin levels in about 60% of PNH patients. However, the required doses of corticosteroid are high, and a long-term continuous administration on a daily basis is difficult due to its toxicities [2,3]. Owing to immunosuppressive effect of corticosteroid, American thoracic society (ATS) for tuberculosis (TB) recommends anti-TB medication in patients used prednisone more than 15 mg/day over a month [4]. Nevertheless, these guidelines show only the treatment principles, in clinical practice, there are some problems to decide the test or management in special groups including hematologic diseases. In addition, the prevalence of miliary TB in malignant disease is 3 times higher than in general population and its mortality rate is about 25–30% in adults [5].

We experienced a PNH patient with acute attack. He received corticosteroid for 3 weeks to control hemolysis. In the past, he was diagnosed with pulmonary TB and we investigated LTBI (latent TB infection). He was not indicated with anti-TB medication [6], but after immunosuppressive drug for 3 weeks, he suffered from miliary TB. So we report this difficult and embarrassing case with literature review of TB associated with hematologic disease.

## Case report

**Figure 1:**
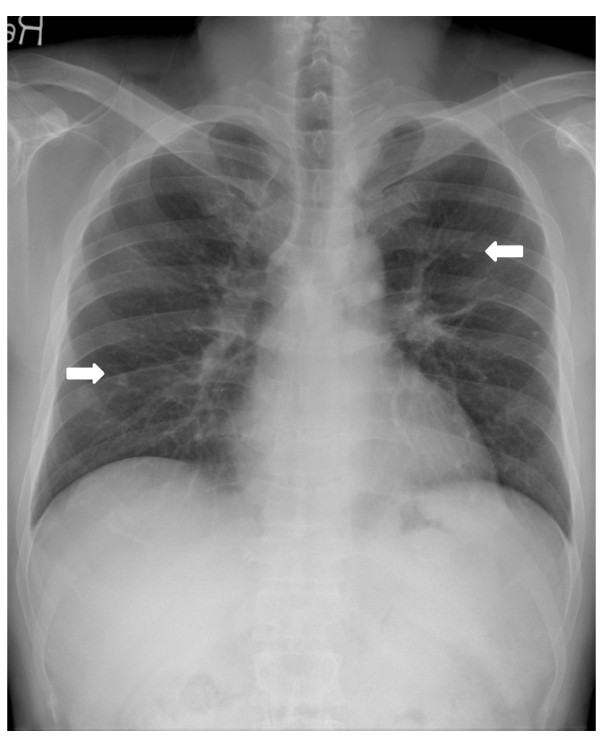
In chest radiographs, the suspicious old tuberculous calcified nodule was seen (white arrow).

**Figure 2:**
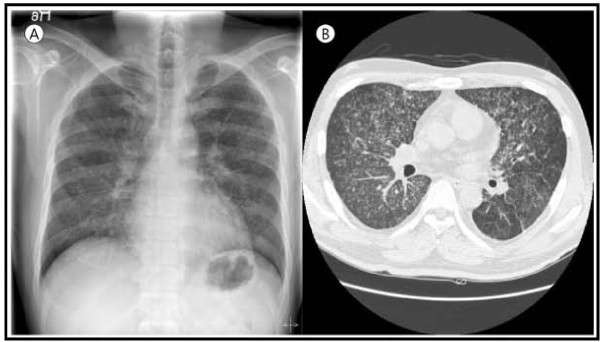
** (A) In chest radiographs, the miliary nodules were observed in both lung field.** ( **B**) Chest CT scan showed miliary nodulation and patch underlying perinodular ground glass appearance in both lung field, suggestive of miliary TB.

**Figure 3:**
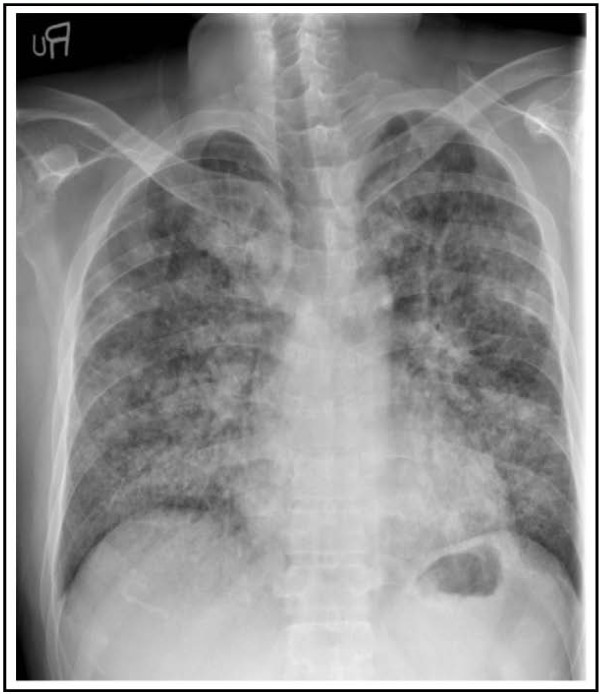
Patch density in chest X-ray was progressed, suggestive of acute respiratory distress syndrome.

**Figure 4:**
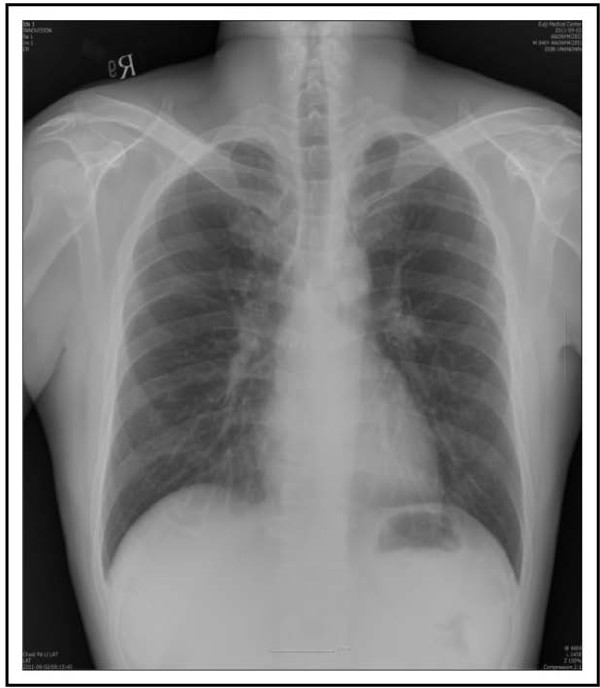
After anti-tuberculosis medication, chest X-ray showed improvement.

A 40-year-old Asian male patient presented with fever and more aggravated generalized weakness for 1 week. A month ago, he complained of hematuria, fever and generalized weakness. He was diagnosed with PNH associated with another bone marrow disorder (aplastic anemia). For control of acute hemolysis, red cells transfusion and immunosuppressive drugs (corticosteroid and danazol) were prescribed and he was discharged after stabilized. At that time, his chest X-ray showed old pulmonary calcified nodules in both lung field (Figure 1). He was diagnosed with pulmonary TB about 21 years ago and he was completely cured after anti-TB medication (Isoniazid, Ethambutol, Rifampicin, Pyrazinamide) for 6 months. On readmission, vital signs were followed; BP 140/80 mmHg, pulse rate 76 beats/min, respiration rate 20 times/min, and body temperature 39.1°C. Jaundice was observed in both sclera, and breath sounds were decreased in both lung field. The laboratory findings revealed white blood cells, 720/uL (segment neutrophil 75.6%; lymphocytes 15.7%); hemoglobin, 9.6 g/dL; and platelets, 55,000/uL. AST/ALT, total bilirubin and LDH were 167/312 IU/L, 3.9 mg/dL and 2,543 IU/L respectively. Blood urea nitrogen and serum creatinine were 20.3 mg/dL and 1.4 mg/dL. In chest radiographs, the miliary nodules were observed in both lung field (Figure 2A). Chest CT scan showed miliary nodulation and patch underlying perinodular ground glass appearance in both lung field, suggestive of miliary TB (Figure 2B). TB PCR was positive. After 3 weeks of immunosuppressive drugs, he was diagnosed to miliary TB. Due to elevated liver enzyme, Ethambutol/Moxifloxacin/Cycloserine were selected. To expect the rise of drug effects, supportive care such as G-CSF administration, blood transfusion and nutritional support were done. After 5 days of the anti-TB medications, patch density in chest X-ray was progressed and he complained of severe dyspnea, nausea, vomiting and aggravated weakness (Figure 3). We considered ventilator care, but the patient was tolerable at the 10L of oxygen mask apply. We added 1^st^ line anti-TB drugs (Isoniazid, Rifampicin) stage by stage. After continuous intensive care for 3 weeks, dyspnea was getting better and he needed only 2L of oxygen apply. The chest X-ray has got better. He was improved and the resistance strain was not seen in 8 weeks after culture. He had taken 1^st^ line anti-TB drugs (Isoniazid, Ethambutol, Rifampicin) and warfarin in outpatient of hospital, and nowadays he finished anti-TB drugs except warfarin (Figure 4).

## Discussion

In paroxysmal nocturnal hemoglobinuria (PNH), the absence of the glycosyl phosphatidylinositol (GPI)-anchored proteins induces hemolytic anemia, thrombosis and smooth muscle dystonias. PNH is a chronic condition and its treatment includes 1) allogeneic bone marrow transplantation, 2) eculizumab, a humanized monoclonal antibody against the terminal complement protein C5 [7,8] and 3) supportive care such as transfusion, iron and folic acid supplementation and prophylactic anti-coagulation. However, sometimes patients have acute attacks. Corticosteroid can alleviate a hemolytic paroxysm and improve hemoglobin levels in about 60% of PNH patients. But a long-term continuous administration of high dose corticosteroid is troubled with its toxicity [2]. Due to immunosuppression of corticosteroid, CDC recommends prophylactic anti-TB medication in patients with more than 15 mg/day prednisone over 1 month [4].

Worldwide, for the elimination of TB, identification and treatment of latent tuberculosis infection (LTBI) are essential [9-12]. LTBI is a condition in which a person is infected with dormant Mycobacterium tuberculosis organisms, but does not currently have active TB disease [13]. According to ATS for TB, following patients with LTBI should be considered for anti-TB medication; HIV infection, organ transplantation related to immune- suppressant therapy, recent TB infection within 2 years, TNF inhibitors. In cellular immunity of TB, lymphocyte is a major part of immune activity [4,14]. In patients with hematologic disease, T-cell immunodeficiency was induced by hematologic disease itself, chemotherapy, corticosteroid and other underlying diseases [15]. Hahn et al. reported that the prevalence of active TB in hematologic disorders was 0.8% in total, with 21.4% in myelofibrosis, 20% in PNH and 7.9% in myelodysplastic syndrome in Korea. Although in this report, the prevalence of TB in the hematologic disorder was not significantly different compared with control group, the incidence of TB in PNH was very high [16]. There are many controversies in the incidence of TB in immunosuppressed patients. In clinical practice, considering risk and an incidence, the diagnosis and treatment of LTBI should be determined. However, there is a problem in determination of prophylactic anti-TB medication, that is to say limitation of the diagnostic methods for LTBI in immunosuppressed patients.

American Thoracic Society (ATS) proposed targeted tuberculin skin test (TST) as a diagnostic tool for LTBI in high-risk group and in the U.S. diagnostic guidelines of 2000, an immunologic test was described. QunatiFERON-TB Gold (QFT-G) is an interferon-gamma releasing assay (IGRA) and it was approved by the FDA for the diagnosis of TB in 2005 and replaced TST [4,17]. In Korea with high prevalence of TB and high false positivity of TST due to BCG vaccination, IGRA replace TST. But IGRA has a limitation in differentiation of active TB from LTBI, and when patient was treated with LTBI or active TB in the past, it can’t determine whether or not new infection is developed. Also in patients with immunosuppression and complete recovery from TB at the same time, there is no guideline.

On the other hand, miliary TB accounts for about 1% of all cases of TB and the mortality related to miliary TB is about 25–30% in adults [5]. Miliary TB may infect any number of organs, including the lungs, liver and spleen. Hematologic abnormalities are frequently described and pancytopenia is profound in hematolgic diseases. In malignant diseases, the incidence of miliary TB is about 3% and it rarely reported in hematologic diseases [18,19].

In our patient, before the administration of immunosuppressive drugs for control of acute hemolysis, we wanted to perform the test for detection of LTBI according to CDC for TB. However, since our patient did not have clinical and radiological evidence of active TB and he was completely cured TB in the past, he was not indicated to test and prophylactic anti-TB medication. In addition, because anti-TB medication frequently induces hepatic dysfunction, poor oral intake, nausea and vomiting, the administration prophylactic anti-TB medication is very troubled in seriously ill patient out of guideline. However, he contracted miliary TB and we were in confusion. The treatment for TB was not easy because he complained of nausea, vomiting and poor oral intake and his laboratory findings showed severe pancytopenia and liver dysfunction. Meanwhile, fever is the sign that needs differential diagnosis. He had fever at diagnosis of PNH due to hemolysis and he was checked chest X-ray regularly (weekly). But, miliary TB was developed in one week. So in patients with fever and immunosuppressive drugs, inspection and chest X-ray at intervals of 2 or 3 days are needed.

In conclusion, except some advanced country, TB is endemic disease in worldwide yet. Also, there are differences of TB prevalence between the subgroups of hematologic diseases. So we emphasize the need to investigate multicentral TB prevalence and to make an urgent decision of details of the guidelines for anti-TB medication in patients with subgroups of hematologic diseases including PNH.

## Consent

Written informed consent was obtained from the patient for publication of this case report and any accompanying images. A copy of the written consent is available for review by the Editor-in-Chief of Annals of clinical microbiology and antimicrobials.

## Competing interests

The authors declare that they have no competing interests.

## Authors’ contributions

JL and SG participated in the concept and design of the manuscript. BL provided pulmonologic concept of this manuscript. All authors read and approved the final manuscript.
